# Estimated glomerular filtration rate in observational and interventional studies in chronic kidney disease

**DOI:** 10.1007/s40620-024-01887-x

**Published:** 2024-02-12

**Authors:** Michele Provenzano, Lilio Hu, Chiara Abenavoli, Giuseppe Cianciolo, Giuseppe Coppolino, Luca De Nicola, Gaetano La Manna, Giorgia Comai, Olga Baraldi

**Affiliations:** 1 Nephrology, Dialysis and Kidney Transplant Unit, IRCCS Azienda Ospedaliero-Universitaria di Bologna Policlinico Sant′Orsola, Bologna, Italy; 2 Department of Medical and Surgical Sciences (DIMEC), Alma Mater Studiorum - University of Bologna, 40138 Bologna, Italy; 3 Renal Unit, Department of Health Sciences, University “Magna Graecia” of Catanzaro, Catanzaro, Italy; 4 Renal Unit, University of Campania “Luigi Vanvitelli”, Naples, Italy

**Keywords:** eGFR, Observational studies, Interventional studies, CKD, ESKD

## Abstract

Estimated glomerular filtration rate is considered the principal measure of kidney function and, together with albuminuria, is a relevant prognostic factor for the development of end-stage kidney disease. Due to the strong association between estimated glomerular filtration rate and clinical events, such as commencement of dialysis, cardiovascular outcomes and all-cause death, estimated glomerular filtration rate is crucial for clinical decision-making in terms of scheduling follow-up and pharmacological interventions, and planning renal replacement therapies in advanced chronic kidney disease. In this review we discuss the available methods for measuring glomerular filtration rate and for estimating it through mathematical equations developed over the last few decades. We summarize the prognostic association of different percentages of estimated glomerular filtration rate decline and the main clinical outcomes, and how treatments modify estimated glomerular filtration rate decline and the risk of future endpoints. We also examine the role of pre-clinical trial slope and that of estimated glomerular filtration rate as a useful biomarker when evaluating patients for inclusion into both observational and interventional studies.

## Introduction

Chronic kidney disease (CKD) is emerging as one of the most prevalent non-communicable diseases, affecting about 850 million individuals worldwide and contributing significantly to cardiovascular (CV) events, mortality and disease progression to End-Stage Kidney Disease (ESKD), the most advanced stage of CKD which significantly increases mortality and affects quality of life [1]. A reliable risk stratification of CKD patients is pivotal to predict the risk of future CV and kidney events and to implement strategies to improve individual prognosis [2]. Glomerular Filtration Rate (GFR) is widely recognized as the best measure of the degree of kidney function, which is proportional to kidney size, that, in turn, is proportional to body surface area (BSA). Moreover, kidney function strongly influences fluid and electrolyte balance, metabolism and elimination of nitrogen products, regulation of acid–base balance and mineral metabolism, and synthesis and secretion of erythropoietin as well [3]. According to the Kidney Disease: Improving Global Outcomes (KDIGO) 2012 Clinical Practice Guidelines, GFR is the main criterion for classifying CKD severity, and its unit of measurement (mL/min) is standardized to a body surface area of 1.73 m^2^ [4]. Glomerular filtration rate category G1 is defined as GFR ≥ 90 mL/min/1.73 m^2^, G2 as GFR 60–89 mL/min/1.73 m^2^, G3a as GFR 45–59 mL/min/1.73 m^2^, G3b as GFR 30–44 mL/min/1.73 m^2^, G4 15–29 mL/min/1.73 m^2^, and G5 as GFR < 15 mL/min/1.73 m^2^ (ESKD); the second main kidney measurement which contributes to risk stratification in the KDIGO classification is albuminuria. Both GFR and albuminuria have complementary roles in predicting kidney disease worsening, onset of CV events and mortality, albeit having a different pathophysiological meaning, being the former a marker of kidney function and the latter the key marker of kidney damage [5–7]. On this background, the use of albuminuria and GFR has rapidly spread to clinical practice. Glomerular Filtration Rate is estimated (eGFR) by different equations mainly using serum creatinine concentrations (mg/dL) and clinical parameters. In healthy subjects, measured GFR normally declines with age by approximately 1 mL/min/1.73m^2^/year beginning from the fourth decade of life [8]. Overall, a fast GFR decline (> 3 mL/min/1.73m^2^/year) is indicative of underlying kidney disease and tendency to progress to ESKD, regardless of age and of many other risk factors [9]. The eGFR threshold that is used to define CKD is < 60 mL/min/1.73 m^2^, confirmed for at least 3 months. Below this level, the clinical course of the disease is characterized by the onset of severe complications, such as arterial hypertension [10], metabolic acidosis, anemia, electrolyte imbalance (hyperkalemia and hyperphosphatemia), metabolic acidosis, mineral bone disorders and systemic atherosclerosis [11]. Although the association of eGFR and poor prognosis has been well established, considerable effort is still needed to define how to properly use this measure in clinical research (and clinical practice as well), especially regarding which levels actually warrant nephroprotection in case of randomized clinical studies, and how to determine which levels herald future cardiorenal risk in prognostic studies.

The purpose of this review is to analyze the role of eGFR in observational and interventional studies, in an effort to establish the meaning of this key kidney measurement, to provide a deeper understanding of the management of CKD patients, and to reinforce the study design in CKD.

## Glomerular filtration rate measurement

Glomerular filtration rate is the main parameter used to assess the degree of kidney function in health and disease and it reflects the sum of nephron filtration rates expressed in terms of milliliters of filtrate per minute (mL/min) as average. The kidneys of a healthy young individual filter approximately 180 L of plasma per day, that corresponds to a GFR level of about 125 mL/min [12]. To date, normal eGFR values are considered to be between 90 and 125 mL/min, whereas values above 125 mL/min may be abnormal and mainly represent the hemodynamic effect of single-nephron hyperfiltration [13]. This is particularly true for diabetic kidney disease (DKD), which is the major cause of ESKD worldwide and defines kidney damage caused by diabetes mellitus. The first stage of DKD is characterized by hyperfiltration, triggered by hyperglycemia and by the deactivation of tubuloglomerular feedback due to hyper-reabsorption of sodium in the proximal tubule. This should be considered when adopting the eGFR in diabetic patients. However, the actual GFR value cannot be measured directly but rather is derived from the calculation of urinary or plasma clearance of filtration markers. Urinary or renal clearance is defined as the ratio between urinary excretion (urinary concentration multiplied by urine flow rate) and plasma concentration of a substance, while plasma clearance is defined as the volume of plasma completely cleared of a substance per unit of time. Knowing these two definitions, the rate of renal clearance of a substance is equal to its plasma removal rate when the substance is not synthesized or metabolized by the kidneys. In the past century, the search for the ideal marker of kidney filtration has highlighted the difficulties in measuring GFR. The ideal filtration marker should: be inert and non-toxic; be excreted exclusively by the kidney; not be metabolized by the kidney; have constant plasma concentration and be easily measured in plasma and urine; not be bound by plasma protein, and be freely filtered through the glomerulus (i.e. molecular weight < 20,000 Da); be neither secreted nor reabsorbed by the tubule [14]. Historically, urinary clearance of inulin, an exogen biomarker, has been the gold standard for measuring GFR since this exogenous substance fulfills all the properties of an ideal filtration marker [15]. However, the classic method for measuring inulin clearance is time-consuming in clinical practice as it requires constant intravenous infusion to achieve a stable plasma level, repeat blood sampling for plasma measurement, and urine collection through bladder catheterization [14]. Therefore, inulin has been replaced by alternative and more convenient exogenous filtration markers for clearance measurements, such as iohexol, iothalamate, chromium ethylenediaminetetraacetic acid (^51^Cr-EDTA) and diethylenetriaminepentaacetic acid (^99m^Tc-DTPA) [16]. A systematic review with meta-analysis of 83 studies comparing different markers to inulin concluded that the most accurate methods for measuring GFR are urinary clearance of iothalamate or ^51^Cr-EDTA and plasma clearances of 51Cr-EDTA or iohexol [17].

In clinical practice, clearance of an endogenous substance, such as creatinine and urea, has been accepted as the mainstay to measure GFR due to the feasibility of measurement. However, serum urea level is also influenced by volemia (higher in volume depletion), it increases in individuals on high-protein diet and glucocorticoid therapy, and urea clearance (Cr_U_) is compromised by tubular reabsorption, which lowers urine urea excretion, therefore underestimating its clearance [18]. Creatinine is a product of muscular metabolism (creatine) and is influenced by muscle mass (higher in young men and in African-Americans) and partially by diet (higher after ingestion of meat). Furthermore, it is partially secreted by renal tubules, thus resulting in an overestimation of GFR when assessing creatinine clearance (Cr_Cl_), especially in patients with advanced kidney disease [19]. In case of very low urine flow rates, creatinine reabsorption may also occur [20]. To overcome the problem derived from the overestimation of GFR and to integrate the individual variability of non-GFR determinants of serum creatinine (age, sex and body surface area), several mathematical equations have been developed over the past decades (Tables 1 and 2).

**Table 1:**
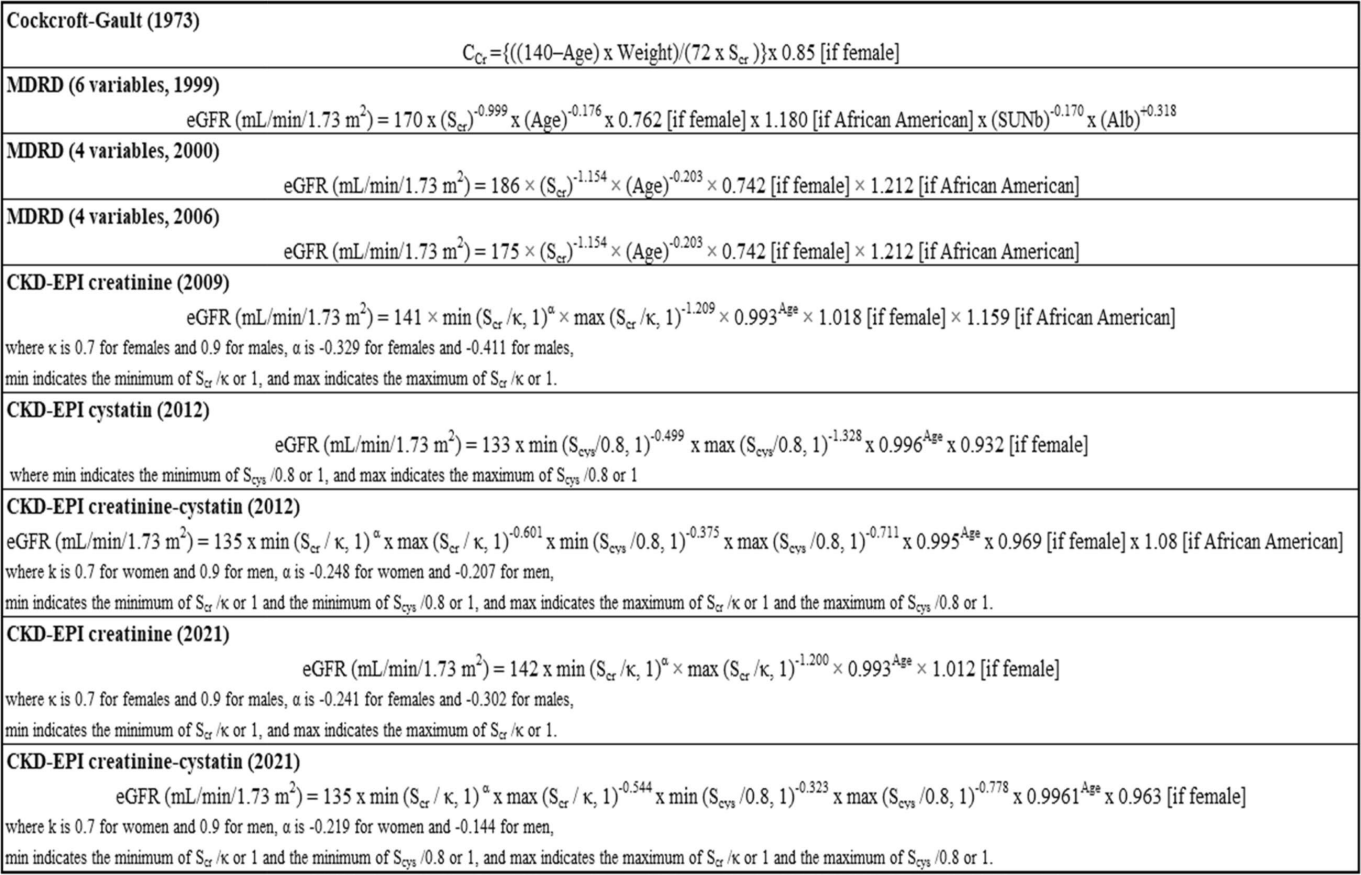
GFR-estimating equations for adults. CCr creatinine clearance, mL/minute

Age, years. Weight, kg

Scr standardized serum creatinine, mg/dL. Scys standardized serum cystatin C, mg/L

**Table 2:**
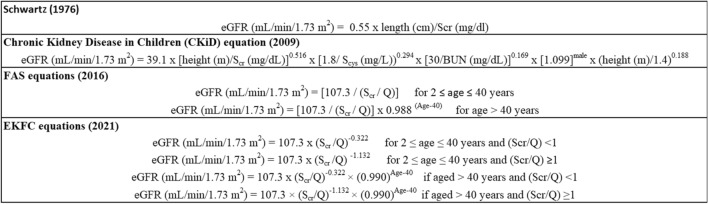
GFR-estimating equations for children and adults

eGFR estimated Glomerular Filtration Rate, Scr standardized serum creatinine, mg/dL. *Q* value corresponds to median serum creatinine value for age-/sex-specific healthy populations

## Estimated GFR equations for adults

### Cockcroft-Gault equation

One of the first formulas developed to predict creatinine clearance was the Cockcroft-Gault formula (CG), that was elaborated (on the basis of 4 parameters: serum creatinine, age, body weight and sex) from data of 236 men ranging in age from 18 to 92 years old and with a measured creatinine clearance of 30–130 mL/min [21]. The Cockcroft-Gault equation had been accepted and widely used since its introduction in 1976, however two considerations are necessary. First, in case of overweight and obese patients, a specific body weight adjustment (using 40% of actual body weight, ABW_0.4_) was needed to calculate a more accurate creatinine clearance, that otherwise, would be overestimated [22]. Second, most clinical practitioners used to round serum creatinine concentration values up to standard values of 0.8 or 1 mg/dL in case of low serum creatinine levels in elderly patients in whom low levels may be secondary to decreased muscle mass; however, many studies have found that such rounding does not improve the performance of the CG equation, and on the contrary, actually results in erroneous underestimation of creatinine clearance [22, 23]. Because the original equation was not expressed using standardized creatinine values, the CG equation has partially lost its importance in the estimation of kidney function. Indeed, CG has been used to adjust the drug dose on the basis of the kidney function, and to date, this continues to be reported in the summary of drug characteristics, although since 2010 the Food and Drug Administration (FDA) has warned the industry to use the Modification of Diet in Renal Disease (MDRD) Study equation as it is a more appropriate option [24].

### The modification of diet in renal disease equation

The MDRD equation was developed in 1999 by Levey et al. from observations made on a population of 1628 patients with predominantly non-diabetic CKD enrolled in the MDRD Study, a multicenter controlled trial that evaluated the effect of dietary protein restriction and blood pressure control on the progression of CKD [25]. The original MDRD Study prediction equation included six variables: age, sex, ethnicity, and serum concentration of creatinine, albumin and urea nitrogen. Subsequently, in 2000, a simplified version of the MDRD Study equation with 4 variables (consisting of age, sex, ethnicity and serum creatinine concentration) was proposed [26]. In 2006, an updated 4-variable MDRD Study equation, re-expressed to integrate creatinine standardization, was introduced [27]. The MDRD Study equation has shown to be superior to the CG equation and it was thus largely used to estimate GFR in clinical practice. The main limitation is the systematic underestimation of higher GFR level (> 60 mL/min/1.73 m^2^) because the equation was developed in patients with overt CKD.

### The chronic kidney disease epidemiology collaboration equation

Therefore, in 2009, the National Institute of Diabetes, Digestive and Kidney Disease (NIDDK) proposed the Chronic Kidney Disease Epidemiology Collaboration (CKD-EPI) equation to estimate GFR. It was based on the same four variables as the MDRD Study equation, with the objective to be as accurate as the MDRD Study equation at GFR < 60 mL/min/1.73 m^2^ and even more accurate at higher GFRs [28]. The equation was developed from an analysis of the demographic and clinical parameters (standardized creatinine concentration, sex, ethnicity and age) of 8254 participants (30% with diabetic CKD) from 10 studies with measured GFR with iothalamate clearance ranging between 2 and 190 mL/min/1.73 m^2^. The equation was then validated on 3896 participants from 16 studies using iothalamate and other filtration markers. The 2009 CKD-EPI equation proved to be less biased as compared to the MDRD Study equation especially in the healthy population with eGFR > 60 mL/min/1.73 m^2^ [29]. In fact, although eGFR cannot replace the direct GFR measurement to evaluate pre-donation kidney function [30], when measured GFR (mGFR) is not available, eGFR values estimated by CKD-EPI should be preferred over those calculated by the MDRD equation to avoid underestimating GFR [31]. An analysis conducted on a cohort of 394 individuals with a median age of 80 years demonstrated that the CKD-EPI equation was more accurate than the MDRD Study equation, even in the elderly [32]. However, due to variations in non-GFR determinants of serum creatinine other than age, sex and race, the formula remained limited due to possible misclassification of patients, with the risk of erroneously classifying patients without CKD (based on GFR measurement) as CKD (at different stage), and viceversa. This is particularly relevant for clinical practice since it increases the risk of adopting unnecessary interventions in terms of diagnosis and therapy or, vice-versa, not implementing necessary interventions in high-risk patients [28]. Current guidelines recommend lowering targets of CV risk factors, avoiding contrast media for imaging procedures, titrating drug doses for drugs that are excreted by the kidney in patients with CKD, and creating an arteriovenous fistula in patients close to dialysis. Therefore, overdiagnosis of CKD may lead to over-aggressive CV risk factor control, withholding important diagnostic procedures, and insufficient drug dosing.

In 2012, the CKD-EPI proposed two new equations for GFR estimation: one equation, referred to as the cystatin C equation (CKD-EPI cys), was based on standardized serum cystatin C alone; the other one used cystatin C combined with standardized creatinine and was referred to as the creatinine-cystatin C equation (CKD-EPI cr-cys) [33]. The two equations were developed based on data of 5352 heterogeneous participants in 13 studies with a very broad range of measured GFR (5 to 198 mL/min/1.73 m^2^) and validated in 1119 participants in 5 other studies. Cystatin C is a low molecular-weight protein that acts as a cysteine protease inhibitor and is produced by all nucleated cells at a constant rate [34]; it cannot be used as a urinary excretion marker since it is normally almost completely reabsorbed and catabolized by proximal tubule cells. However, it can be used as an alternative filtration marker for estimating GFR, though more accurate than the creatinine-based estimates in specific cases since the value is less affected by diet and muscle mass [35]. According to CKD-EPI 2012, cystatin C should not replace creatinine in GFR estimates in general practice due to higher costs and less availability; indeed, cystatin C-based equations may be used as a confirmatory test to diagnose CKD in patients with a decreased estimation of GFR from creatinine [33]. However, higher cystatin C levels are related to current smoking, male sex, increased body mass index with high total body fat and lower lean body mass, and higher C reactive protein levels [36]. The combined creatinine-cystatin C equation showed better accuracy than equations based on either creatinine or cystatin alone (percentage of estimates that were > 30% of measured GFR were 8.5% vs. 12.8% and 14.1%, respectively): the improved precision can be linked to a smaller influence of non-GFR determinants of creatinine and cystatin C in a formula that uses both markers [33].

In 2020 the National Kidney Foundation (NKF) and the American Society of Nephrology (ASN) established a task force to reassess the inclusion of race in the estimation of GFR, due to the fact that race is a social and not a biologic construct [37]. In 2021, the CKD-EPI proposed new eGFR equations without race, developed from two data sets of 8254 participants (31.5% Black) from 10 studies for serum creatinine and 5352 participants (39.7% Black) from 13 studies for both serum creatinine and cystatin C. The equations were validated on a data set of 12 studies including 4050 participants, 14.3% Black. The comparison of the accuracy of the new creatinine equations omitting race showed increased estimates of CKD prevalence among the Black population with underestimation of GFR (median, 3.6 mL/min/1.73 m^2^; 95% CI 1.8–5.5) and lower prevalence among non-Blacks with overestimation of GFR (median, 3.9 mL/min/1.73 m^2^; 95% CI 3.4–4.4). The new CKD-EPI cr-cys equations without race proved to be more accurate than the new creatinine or cystatin alone equations (38).

### Considerations about estimated GFR formulas

The advantage of the 2021 CKD-EPI equations that omit race among the determinants of serum creatinine is that they are more generalizable across populations, without social and racial implications, thereby facilitating the use of eGFR in clinical practice and public health programs. However, a recent analysis conducted by Delanaye et al. showed that the performance of the 2021 CKD-EPI equation was still suboptimal in European and African cohorts, generating concerns around its generalizability and reproducibility [39]. Indeed, in a position statement the European Federation of Clinical Chemistry and Laboratory Medicine (EFLM) recommended not implementing the 2021 CKD-EPI equation in European laboratories while keeping the 2009 CKD-EPI version until global consensus is reached [40].

The NKF also rediscussed the accuracy of indexing the eGFR value to a standard BSA. Body surface area may be estimated knowing weight and height [41], and adjusting for BSA is important when assessing eGFR to define the stages of CKD and to allow comparison among individuals with different body size. However, in some circumstances using the indexed BSA of 1.73 m^2^ (this value is the average surface area for an adult with a height of 170 cm and weight of 63 kg) in people with extreme body sizes, with very large BSA or very small BSA, is not correct. In fact, in people with large BSA (obese or tall), the indexed eGFR value is lower than non-indexed eGFR (absolute eGFR adjusted for patient’s BSA), while indexed eGFR is greater than non-indexed eGFR in short and thin people with small BSA. Considering the key role of drug volume of distribution (that is determined by BSA) in the assessment of drug dosage, drug dosing decisions are generally based on non-indexed eGFR in mL/min. Non-indexed eGFR can be computed by multiplying the indexed eGFR value by the patient’s BSA and dividing by 1.73 m^2^ [42].

The main advantage of eGFR is its easy and broad application in clinical practice by most clinicians of different specialties, as compared to measured filtration rate; at the same time the estimate itself remains its main limit. Significant differences between mGFR and eGFR as measured by either MDRD or CKD-EPI have been reported and are definitely non negligible [43]. Indeed, about 25% of subjects with eGFR < 15 mL/min/1.73 m^2^ (theoretically requiring dialysis or kidney transplant) showed a measured GFR of 15–29 mL/min/1.73 m^2^, meaning that a remarkable percentage of patients that are believed to have reached ESKD actually have not yet done so, leading to potential erroneous clinical decision-making. Vice-versa, about 10% of patients are mistakenly classified as having moderate CKD according to eGFR and correctly reclassified as severe CKD according to mGFR, that is, a potential cause of underestimation of risk in clinical practice. Moreover, in some cases of pathologic hyperfiltration as seen in the early stages of DKD [44], classifying CKD using eGFR alone might not be useful. Therefore, nephrologists should not make important decisions based solely on eGFR but should carefully consider the patient’s medical history, other laboratory data and the presence of uremic symptoms.

## Estimated GFR equation for children and adolescents

In 1976, Schwartz et al. developed a simple creatinine-based formula to estimate GFR in children using body height and *k*-values (bedside Schwartz equation), which are constant coefficients dependent on age and sex [45]. Statistical analysis of the resulting eGFR values compared to clearance data in a group of 223 children showed good agreement with creatinine and inulin clearances, with relative correlation coefficients of 0.935 and 0.905, respectively. Due to the evolution in serum creatinine assay kits with enzymatic techniques, this equation was updated in 2009 into the Chronic Kidney Disease in Children (CKiD) equation in a cohort of 349 children aged from 1 to 16 years old and using a fixed *k*-value = 0.413 as a constant coefficient [46]. The inclusion of height, gender, serum cystatin C and urea nitrogen, besides serum creatinine, improved the precision and accuracy of the formula, with 87.7% of eGFR values within 30% of iohexol-measured GFR and 45.6% of eGFR within 10% of iohexol-measured GFR. However, systematic biases with the fixed *k*-value CKiD equation were identified due to the systematic decrease in eGFR for healthy children who follow the average growth curves and have the ideal average creatinine value that is specific to age and gender. Subsequently, in 2021, the CKiD equation was optimized by introducing age-/sex- dependent *k*-values valid across the full pediatric age spectrum and into young adulthood under 25 years (CKiDU25 equation) [47]. The CKiDU25 equation overcomes the limits regarding the age-dependent decline, however an analysis conducted by Pottel et al. showed that this equation is associated with a systematic difference of about 10% between healthy male and female children and adolescents over the entire age range [48]. In children without CKD, the Full Age Spectrum (FAS)-Height [49] and European Kidney Function Consortium (EKFC) equation (50) (see below) may be preferable because of the absence of age and sex effects.

## Full age spectrum equation to estimate GFR

A significant limitation of the MDRD and CKD-EPI equations was the assumption that kidney function starts to decline from the age of 18 years (due to inclusion of age coefficients in the formulas), while mGFR remains substantially unchanged until the age of 40 years [8]. In order to correct this flaw and also to provide continuity with aging that was missing in the available separate equations (Schwartz and FAS-Height equations for children [45, 49], CKD-EPI equation for adults [33] and BIS1 equation for the elderly [51]), a FAS equation was developed by Pottel et al. in 2016 as a valid alternative for estimating GFR for all ages, solving the discontinuity and physiologically implausible eGFR changes when switching from pediatric age to adulthood and from adulthood to older age [52]. However, the FAS equation was limited due to its overestimation of eGFR when serum creatinine was low, therefore, in 2021 the EKFC adopted data from European cohorts to develop an alternative serum creatinine-based eGFR (EKFC eGFRcr) equation (50) that combined features of the FAS equation (52) and the CKD-EPI equations (33). To improve the accuracy of eGFR assessment, a cystatin-based EKFC (EKFC eGFRcys) equation was subsequently proposed [53]. Compared to the 2021 CKD-EPI equation, the EKFC equations showed better performance in European and African populations in all age ranges [39].

## Estimated GFR in observational studies

Observational studies are traditionally designed to investigate the prognostic role of one or more variables in patients under standard-of-care treatment or in a peculiar setting with particular characteristics (e.g. patients followed by nephrologists or patients at increased CV risk). In these studies, eGFR plays a fundamental role as a predictor of clinical events, such as dialysis start, CV outcomes and death. To date, the prognostic role of eGFR for ESKD has been analyzed in several studies [5, 7, 54–56]. All these studies have, overall, demonstrated that eGFR is an instrumental variable in models designed to predict kidney and CV endpoints. Recently, a multicenter study evaluated long-term renal prognosis in 757 first kidney transplant recipients from deceased donors, compared to 1940 patients with native CKD followed by nephrologists [54]. The aim of this original investigation was to compare risk and determinants of ESKD between the two cohorts. In both cohorts, eGFR was significantly associated with ESKD risk (with a *p* value < 0.001). When the hierarchy of prognostic factors was tested, eGFR proved to be the greatest contributor in the kidney transplant recipient group (19.1%) and in the native CKD group (48.9%). According to the prognostic model developed by the Authors, the prognostic weight of eGFR estimated at 1 year after transplantation was higher than the main ESKD determinants specific to kidney transplantation: delayed graft function (17.9%), dialysis vintage (17.4%), history of acute rejection occurring in the first 12 months of transplantation (7.4%) and use of calcineurin inhibitors (0.2%). In the native CKD group, age, male sex and smoking contributed to ESKD by 7.7%, 2.0% and 0.2%, respectively. The prognostic role of eGFR is higher versus other variables in high-risk patients, such as kidney transplant recipients and CKD patients referred to nephrologists. In the general population, eGFR still plays an important predictive role whereby the strongest predictor is the presence of proteinuria (or albuminuria) which is a marker of kidney damage. Tangri and colleagues developed a 4-variable risk equation (KFRE) demonstrating that the combination of age, gender, eGFR and albuminuria reached a great discrimination power (c-statistic of 0.91) and that the addition of any other variables did not improve the discrimination of future events prediction [57]. The role of eGFR on renal prognosis was also assessed in larger and more heterogeneous cohorts. In 2011, a large meta-analysis conducted by the Chronic Kidney Disease Prognosis Consortium, evaluating 9 cohorts with 845,125 individuals from the general population and 8 cohorts including 173,892 individuals at high risk for CKD, demonstrated that lower eGFR and higher albuminuria were associated with higher risk for ESKD in both populations [55]. The risk for ESKD in the general population cohorts was unrelated to eGFR at values ranging from 75 to 105 mL/min/1.73 m^2^ and increased exponentially at lower eGFRs. Hazard ratios of ESKD (95% confidence interval) at eGFR levels of 60, 45, and 15 mL/min/1.73 m^2^ (versus 95 mL/min/1.73 m^2^) were 3.69 (2.36–5.76), 29.3 (19.5–44.1) and 454.9 (112.4–1840.2), respectively. An additional important finding was that eGFR and albuminuria were associated with ESKD risk, regardless of conventional cardiovascular risk factors and regardless of each other: the associated risk for low eGFR was similar across all levels of albuminuria and high albuminuria-associated risk was similar across all levels of eGFR. Consequently, these findings were of pivotal importance for subsequent guidelines [4] because they suggested that UACR could be used for stratification of risk for kidney outcomes at any level of eGFR, and that stage 3 CKD (eGFR 30–59 mL/min/1.73 m^2^) [58] may be appropriately subdivided into two stages (eGFR 30–44 mL/min/1.73 m^2^ and eGFR 45–59 mL/min/1.73 m^2^) due to higher risk at lower eGFR level (less than 45 mL/min/1.73 m^2^).

Although the independence of albuminuria and eGFR in the prediction of ESKD has been demonstrated, an effect modification between these two factors has also been proposed and is still the subject of a challenging and interesting debate. Such ‘interaction’ can be explained by pathophysiologic mechanisms. Indeed, proteinuria depends not only on the extent of kidney damage (filtration barrier disease) but also on the residual nephron mass or function: the level of proteinuria reflects the number of filtering nephrons and the capacity of the tubules to reabsorb filtered protein [59]. A low proteinuria level can therefore be a consequence of low eGFR, losing its prognostic significance of a better outcome [60]. Indexing 24-h proteinuria (Uprot) to eGFR value (filtration-adjusted proteinuria, F-Uprot), calculated as Uprot/eGFR × 100, may provide a more precise estimate of proteinuria-induced damage on residual nephrons. A multicenter prospective study conducted by the Collaborative Study Group on the Conservative Treatment of CKD of the Italian Society of Nephrology evaluated the strength of association of F-Uprot with ESKD risk, compared to absolute 24-h proteinuria [5]. The results showed that F-Uprot had a stronger prognostic role on ESKD across all stages of advanced CKD, as it was overall associated with a net reclassification improvement of 12.2% (95% CI 4.2–21.1) (12.2% of patients were reclassified correctly for ESKD risk by F-Uprot when this replaced Uprot), especially in elderly, CVD, diabetic, glomerulonephritis and CKD stage G4 and G5 patients. This finding may open novel questions for observational (and also interventional studies, see below) research, namely the inclusion of F-Uprot or its change over time as a prognostic biomarker in different stages of CKD although this still needs to be confirmed.

The interaction effect of eGFR with other variables needs to be considered. For example, the prognostic impact of eGFR levels change by age strata [61, 62]. It has been demonstrated that age acts as an effect modifier on ESKD and mortality risk, being the risk of ESKD higher than mortality for patients < 60 years old, for almost all eGFR levels. Conversely, risk of death exceeds ESKD risk in older CKD patients (> 65 years) for eGFR levels around 35 mL/min or higher [63]. Hence, in the context of patients with higher eGFR levels, other risk factors, especially the degree of albuminuria (or proteinuria) may improve the prediction of future events. This can also help in the future classification of CKD which currently does not consider age as a classification criterion.

With respect to CV events, the prognostic role of eGFR (i.e. coronary disease, stroke, heart failure, cardiovascular mortality) confirmed the previous findings of kidney risk. A large meta-analysis conducted by the CKD Prognosis Consortium on 637,315 individuals without a history of CV disease from 24 cohorts (19 general-population cohorts, 3 high-CV risk cohorts consisting of diabetic patients, and 2 cohorts of CKD patients) [7] showed that CV outcomes in a 5-year timeframe were substantially constant for eGFR between 75 and 105 mL/min/1.73 m^2^, and increased for eGFR < 75 mL/min/1.73 m^2^, with a steeper risk gradient in particular for heart failure and CV mortality than for stroke and coronary disease. Even more important, the concordance statistics (C-statistics) for CV outcomes based on single traditional risk factors (such as diabetes, smoking, dyslipidemia, and hypertension) ranged from 0.729 to 0.838 in non-CKD cohorts, and showed that there was improvement in discrimination when either or both eGFR and albuminuria were added. Despite the broader implications of albuminuria for CV prediction compared to eGFR (C statistic difference for CV mortality was 0.0139 [95% CI 0.0105–0.0174] for albuminuria and 0.0065 [0.0042–0.0088] for eGFR, C statistic difference for heart failure was 0.0196 [0.0108–0.0284] for albuminuria and 0.0109 [0.0059–0.0159] for eGFR), eGFR still remains a useful predictor of CV events. In fact, in individuals with CKD the combination of eGFR and albuminuria significantly outperformed any single modifiable traditional risk factor for all cardiovascular outcomes: C statistic difference was 0.0227 [0.0158−0.0296] compared to less than 0.007 for any single traditional predictor.

The causal link between eGFR reduction and increased cardio-renal risk is not simple to explain in terms of pathophysiological mechanisms. Worsening of renal function is accompanied by extracellular volume expansion and dependent hypertension (if salt intake is not reduced), a significant risk of atherosclerotic vascular damage as well as by an increase in blood levels of uremic toxins [64]. In keeping with this hypothesis, the Japanese Hisayama Study demonstrated the association of low eGFR with severity of coronary atherosclerosis in population-based autopsy samples [65]. Nakano and colleagues showed the gradual progression of coronary atherosclerosis with calcified lesions, even in patients with moderate stages of CKD, emphasizing the importance of managing CV risk factors before patients reach advanced CKD. This association may be explained by the fact that lower eGFR is associated with increased levels of novel CV disease risk factors, including inflammation, anemia, abnormal calcium-phosphate metabolism, and oxidative stress [66], in addition to traditional CV risk factors (age, hypertension, dyslipidemia, diabetes) [67].

**Fig. 1:**
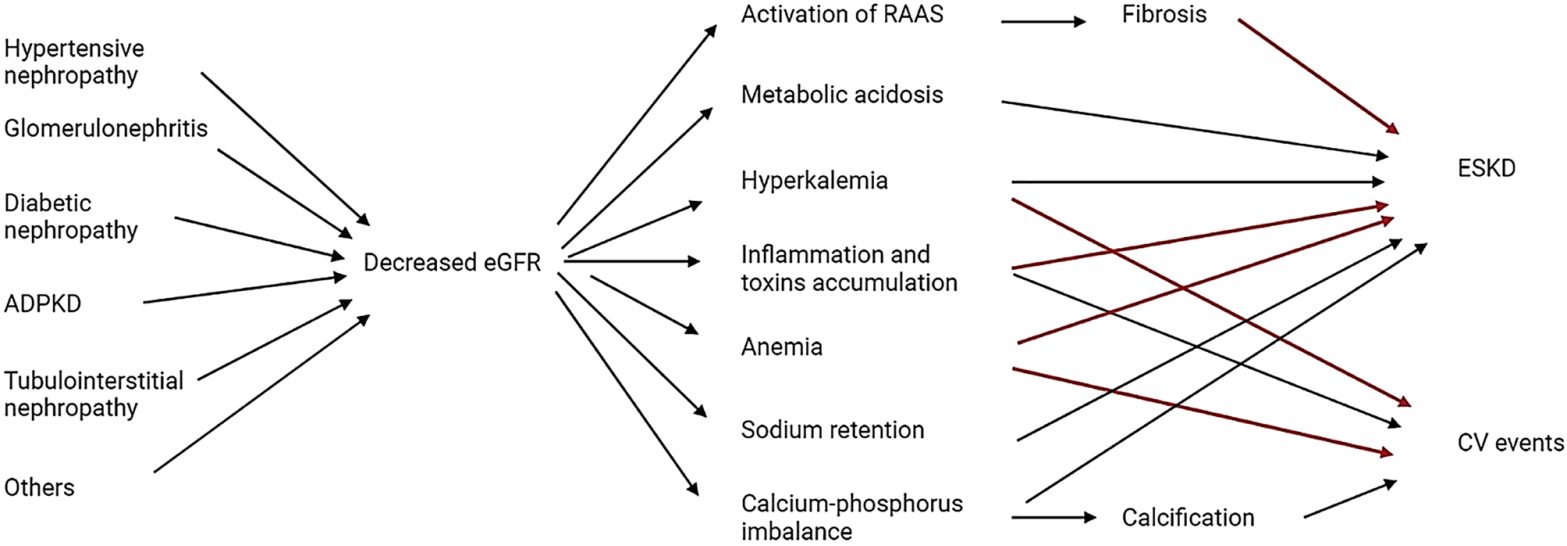
Direct acyclic graphs depicting the role of estimated Glomerular Filtration Rate (eGFR) decline, comorbidities and other conditions which cause the increased risk of cardiovascular (CV) events and progression to end-stage-kidney-disease (ESKD), Autosomic Dominant Polycystic Kidney Disease (ADPKD), Renin–angiotensin–aldosterone system (RAAS). The colors of the arrows refer to the strength of the association between two variables, with the strongest relationships being represented in red

Another important feature of eGFR in observational research is study duration. Normally, hard endpoints such as ESKD require a long time to be reached, even a decade or more. Hence, the development of surrogate endpoints has been solicited to shorten the study duration and to capture a greater number of events which allow a higher number of predictors to be included in the prognostic models. This led to the development of alternative endpoints for CKD progression. The surrogate endpoint of doubling of serum creatinine, corresponding to a 57% reduction of eGFR using the CKD-EPI 2009 creatinine equation (28), was a late event and was, in fact, rarely seen over a 1–3 year period. In 2014, a systematic evaluation across 35 cohorts in the CKD Prognosis Consortium including up to 1.7 million participants (with 12,344 ESKD events and 223,944 deaths) documented that smaller eGFR reductions (less than 57%) from baseline were strongly and consistently associated with risk of ESKD [56]. In fact, among cohorts with individuals reaching ESKD, the prevalence of changes in eGFR of − 30% over 2 years was much higher (52% of ESKD cases) than eGFR declines of 57% (16% of ESKD cases); similarly, in terms of mortality cases, a cumulative prevalence of eGFR reduction of 30% was 7.1% vs 0.97% of cases with eGFR change of − 57%. Although the adjusted hazard-ratio of all-cause mortality was higher with greater change in eGFR (HR 1.8 for 30% decline, 3.7 for 57% change), in terms of percent population attributable risk (%PAR) a higher prevalence of smaller eGFR declines exceeds the corresponding lower relative risk (%PAR peak around 30 to 20% decline). The results of these analyses led us to consider a 30% reduction of eGFR (corresponding to a 1.3-fold increase in creatinine level) over 2 years as a valid surrogate endpoint for progression of CKD and mortality [56, 68]. The biological and clinical associations of eGFR reduction and major clinical outcomes are depicted in Fig. 1.

## Estimated GFR in interventional studies

An interventional study, commonly referred to as a clinical trial, tests an intervention (i.e. a new potential drug, a medical device or a procedure) to prove its safety and efficacy in humans before receiving approval. Investigating the efficacy of new treatments in large-scale trials requires the use of clinically meaningful end points. In the case of CKD, disease course is characterized by a variable decline in the eGFR [69]. In the most advanced stage of CKD (i.e. for eGFR below 15 mL/min), the incidence of comorbidities is striking, and therefore resorting to renal replacement therapy is almost immediate. At the same time, the need for renal replacement therapy, regardless of the technique, is considered a major change in the quality of life. Owing to this evidence, ESKD has been used as a hard endpoint in almost all pivotal clinical trials in CKD. Similarly, fatal and non-fatal CV events and mortality are considered major endpoints as well. However, as stated above, kidney failure and the start of renal replacement therapies are late events that in many cases take years to develop, leading to the need for more acceptable endpoints (the so-called surrogate endpoints) to assess drug safety and efficacy within an acceptable period of time. Hence, alternative, reliable events have been considered. In this context, different percentages of eGFR decline after the start of treatment (such as 20%, 30%, 40%, 57%) as clinical trial endpoints have been at the center of interesting analyses over the past years [68, 70, 71]. In order to obtain a sufficient number of events, a 57% reduction of eGFR has been found to be the most appropriate in clinical trials enrolling patients with advanced stages of CKD or in those with rapid progression of kidney disease. The workshop sponsored by the NKF and the US Food and Drug Administration [68] also supported the use of smaller eGFR reductions (e.g. 30% or 40% decline from baseline) as surrogates for ESKD in clinical trials of CKD. A 40% eGFR decline has been validated as a reliable endpoint when some other trial features are respected. In particular, this holds true in studies of at least 2 years (to allow collection of a sufficient number of events) and with a large number of patients involved (at least 1000 patients). Conversely, such a surrogate endpoint should not be used in trials where interventions influence the non-GFR determinants of serum creatinine (e.g., affecting creatinine generation or tubular secretion). One main example, given the novelty of the topic, could be diet-based interventional studies. In these cases, since diet is a factor that modifies body composition and blood creatinine level, it would be more convenient to measure GFR using filtration markers rather than estimating formulas. The combination of hard end-points such as death and ESKD can also help increase the study power and test the study hypothesis [72]. In the setting of high competing risk, for example with older populations, death preceding ESKD can be a major confounder of study findings. In these cases, adopting composite end points, including mortality and other important clinical outcomes, remains a more useful strategy. Lastly, yet equally significant, acute effects of the drug on eGFR values may be a cause of attenuation of the treatment effect on the surrogate end point, this requiring a larger eGFR decline (at least 40% rather than 20 or 30%) as the endpoint itself. In the post-hoc analysis of two multinational randomized controlled trials (Reduction of End Points in Non–Insulin-Dependent Diabetes With the Angiotensin II Antagonist Losartan [RENAAL] Study [73] and Irbesartan Diabetic Nephropathy Trial [IDNT] [74]), Lambers Heerspink et al. investigated whether adopting alternative end points defined by smaller decreases in eGFR would have improved the statistical power of these trials due to the increased number of patients reaching end points [75]. Despite the improved precision of the treatment effect observed with a higher number of endpoints, the authors also detected an attenuation of the magnitude of the treatment effect; this may be explained by the fact that losartan and irbesartan exert an acute effect on eGFR (reduction during the first month of therapy) in opposite direction to the chronic effect (slowing of the eGFR decline rate in the following years). The findings of the post-hoc analysis suggest that adopting smaller declines of eGFR (< than 57%) as end point might not improve statistical significance (z-scores not less than -1.96) in the setting of drugs that exert acute effects on eGFR. Optimistically in these circumstances, when using the 3-month follow-up visit eGFR as the baseline rather than the pre-randomization value, statistical significance may be achieved by using smaller eGFR thresholds with shorter follow-up intervals (*z*-scores for 40%, 30%, and 20% eGFR decline were comparable to 57% eGFR decline).

In addition to its use as an endpoint in clinical studies, eGFR levels are crucial for inclusion of patients in trials on CKD (enrichment biomarker). From a pharmacological perspective, this is a very important point, since many drugs are largely excreted by the kidneys and, therefore, eGFR is an essential inclusion criterion to assess safety and efficacy of new treatments at different eGFR levels [76]. Kidney function level as an inclusion criterion can be assessed once at baseline (for example at a pre-screening or screening visit) [77] or evaluated several times during the run-in period before randomization [78]. However, serum creatinine level and eGFR show short-term intra-individual variation, even day-to-day (due to changes in diet, exercise or hydration), that is unlikely to impact the individual risk of kidney outcome but that contributes to high rates of screening failure in clinical trials (resulting in a waste of time and effort by both medical professionals and patients). In fact, if the eGFR inclusion criterion is 45–60 mL/min repeated in two consecutive visits, a patient whose first result shows 46 mL/min but 42 mL/min at the second visit would initially be eligible for the study and then definitively excluded from it. A screening approach with less stringent eGFR thresholds has been proposed in a post-hoc analysis of the Aliskiren Trial in Type 2 Diabetes Using Cardiovascular and Renal Disease Endpoints (ALTITUDE) study [79]. Waijer et al. determined that simplifying the eGFR inclusion criteria (e.g., from eGFR cut-off of 30–60 mL/min/1.73 m^2^ to 30–75 mL/min/1.73 m^2^ and to 30–90 mL/min/1.73 m^2^ at the confirmation visit after screening) resulted in a higher number of eligible participants and in fewer screening failures (decrease of 85% and 91%, respectively), without a significant decrease in event rates per year (from 9.3 [8.0–10.7] to 9.1% [7.9–10.5] and 9.1% [7.9–10.4], respectively) or an increase in trial duration. Therefore, broadening the eGFR cut-off criteria may be a strategy for enrolling more participants into clinical trials over a shorter time period, thus improving the efficiency of clinical trials. With respect to eGFR as an enrichment biomarker, another point raised concerns among investigators, namely the validity of selecting patients on the basis of the eGFR of a defined, and often short, period (1 or 2 weeks). Moreover, in order to accumulate sufficient clinical end points, most clinical trials in CKD enroll patients with low eGFR levels at risk of progression to ESKD. However, completely ruling out patients without progressive decline of kidney function is not feasible. One option may be evaluation of the pre-trial eGFR slope to identify patients with progressive CKD. In a post-hoc analysis from the Study Of diabetic Nephropathy with AtRasentan (SONAR) trial, the Authors prompted a challenging proof about the pre-trial eGFR as a helpful measure to select participants for the trials [80]. Atrasentan, a selective endothelin receptor antagonist, has been shown to reduce the risk of renal events in patients with type 2 diabetes at risk for ESKD [81]. The Authors analyzed the pre-trial eGFR decline in 630 patients (12.3%) from the total cohort and observed that only 41% of participants had a rapid eGFR decline (at least 5 mL/min/1.73 m^2^ per year) before enrollment into the trial, while the remaining 59% had a relatively stable eGFR trajectory (eGFR decline of 1–5 mL/min/1.73 m^2^ per year or less). Moreover, the analysis showed that the efficacy of atrasentan in slowing progressive kidney function loss was higher in participants with a more precipitous pre-intervention eGFR slope (mean eGFR decline rates in the atrasentan and placebo groups were 3.3 mL/min/1.73 m^2^ and 4.9 mL/min/1.73 m^2^, respectively) [80] (Table 3). These results support the hypothesis that pre-intervention eGFR decline trajectory may be a useful inclusion criterion to better select participants for clinical trials so as to avoid the enrollment of patients with stable CKD who are unlikely to reach ESKD within the duration of a trial. In current research, this criterion is already being used. Our group will collaborate with the large Personalized Drug Response: Implementation and Evaluation in CKD (PRIME-CKD) project funded by the European Union [82], a global study that will provide a comprehensive evaluation of individualized and biomarker-guided treatment of CKD patients. In the core study of the PRIME-CKD project, a clinical study will consider pre-trial slope as an inclusion criterion, among others. Table 3 summarizes the multiple involvement of eGFR in clinical studies.

**Table 3:** Main types of eGFR used in observational and interventional studies

Type of biomarker	Description
Prognostic biomarker	Low eGFR strongly predicts CV events, mortality and ESKD in disparate setting of patients (patients referred to nephrologists, high risk patients, general population cohorts) eGFR decline over time is a surrogate endpoint of CKD progression to ESKD
Enrichment biomarker	eGFR level is used as a biomarker to include patients in both observational and interventional studies More flexible levels of eGFR before randomization may shorten clinical trial duration
Treatment response biomarker	eGFR decline in response to nephroprotective treatment is a realiable surrogate endpoint of ESKD

## Unmet needs and conclusions

The description and implementation of eGFR is a never-ending story. Clinicians may have the impression that everything around it has already been written, confirmed and stated. On the other hand, based on this analysis we can also show the opposite: everything is still ongoing. Debate around the current definition of CKD that does not consider the physiological decline of eGFR with aging is still hot. Using the same fixed eGFR threshold for all ages in the definition of CKD (< 60 mL/min/1.73 m^2^) may result in overestimation of the burden of CKD in the elderly population, with consequent overdiagnosis and potential harm due to unnecessary treatments and procedures. A study by Liu et al. [61] demonstrated that an alternative definition of CKD based on eGFR cut-off points specific for age (< 45 mL/min/1.73 m^2^ for those aged ≥ 65 years, < 60 mL/min/1.73 m^2^ for those aged 40–64 years, and < 75 mL/min/1.73 m^2^ for those aged < 40 years [83]) determines a substantial reduction in the number of subjects affected by CKD. Elderly individuals with isolated mild to moderate reductions in eGFR who were reclassified as having normal kidney aging did not have a higher risk of death and ESKD compared to controls without CKD. Although this evidence is relevant, it is likely that a correct overview of the problem needs to incorporate the assessment of albuminuria or proteinuria. Moreover, the role of eGFR needs to be further investigated in prognostic and predictive studies. As main examples, prognostic studies should clarify how often and when to measure eGFR for the correct risk stratification of patients, whereas in interventional studies eGFR reduction should be confirmed as a valid endpoint of clinical studies; time will tell for both these aims. Since the surrogate endpoints based on the percentage eGFR decline from baseline have some intrinsic limitations, novel simplified measurements of GFR could be considered in clinical studies in the future as endpoint biomarkers [17, 84]. These methods are based on a single injection of an exogenous biomarker (e.g. iohexol) and few repeated blood samples to reach the measured GFR, and have been developed to facilitate the monitoring of kidney function in pre-clinical studies. Potentially, they can also be adopted in clinical studies, in phase II studies at least, to more accurately evaluate treatment response. Furthermore, the pathophysiologic mechanisms that link low eGFR to poor prognosis should be better clarified in the future as well as in ongoing interventional studies. Observational analysis involving novel biomarkers may be of help.
